# Deep learning for large scale MRI-based morphological phenotyping of osteoarthritis

**DOI:** 10.1038/s41598-021-90292-6

**Published:** 2021-05-25

**Authors:** Nikan K. Namiri, Jinhee Lee, Bruno Astuto, Felix Liu, Rutwik Shah, Sharmila Majumdar, Valentina Pedoia

**Affiliations:** Department of Radiology and Biomedical Imaging and Center for Intelligent Imaging, University of California, San Francisco, 1700 Fourth St, Suite 201, QB3 Building, San Francisco, CA 94107 USA

**Keywords:** Osteoarthritis, Machine learning

## Abstract

Osteoarthritis (OA) develops through heterogenous pathophysiologic pathways. As a result, no regulatory agency approved disease modifying OA drugs are available to date. Stratifying knees into MRI-based morphological phenotypes may provide insight into predicting future OA incidence, leading to improved inclusion criteria and efficacy of therapeutics. We trained convolutional neural networks to classify bone, meniscus/cartilage, inflammatory, and hypertrophy phenotypes in knee MRIs from participants in the Osteoarthritis Initiative (n = 4791). We investigated cross-sectional association between baseline morphological phenotypes and baseline structural OA (Kellgren Lawrence grade > 1) and symptomatic OA. Among participants without baseline OA, we evaluated association of baseline phenotypes with 48-month incidence of structural OA and symptomatic OA. The area under the curve of bone, meniscus/cartilage, inflammatory, and hypertrophy phenotype neural network classifiers was 0.89 ± 0.01, 0.93 ± 0.03, 0.96 ± 0.02, and 0.93 ± 0.02, respectively (mean ± standard deviation). Among those with no baseline OA, bone phenotype (OR: 2.99 (95%CI: 1.59–5.62)) and hypertrophy phenotype (OR: 5.80 (95%CI: 1.82–18.5)) each respectively increased odds of developing incident structural OA and symptomatic OA at 48 months. All phenotypes except meniscus/cartilage increased odds of undergoing total knee replacement within 96 months. Artificial intelligence can rapidly stratify knees into structural phenotypes associated with incident OA and total knee replacement, which may aid in stratifying patients for clinical trials of targeted therapeutics.

## Introduction

Osteoarthritis (OA) develops through heterogenous pathophysiologic pathways, a primary reason there are not yet regulatory agency approved disease modifying OA drugs (DMOADs) to date^1–3^. Several studies have recruited large numbers of participants and collected magnetic resonance imaging (MRI) to investigate mechanisms of OA development and classify structural phenotypes^4–6^. MRI serves an important role in quantifying tissue-specific biomarkers and visualizing morphological changes; however, radiographs are typically used for clinical OA diagnosis. In a research context, several studies that utilize MRI are available^7–9^. These research studies are limited and underutilized due to time and expense of high-quality manual image evaluation by trained radiologists.

Rapid OsteoArthritis MRI Eligibility Score (ROAMES) was introduced to stratify knees into structural phenotypes representative of underlying pathophysiologic changes and simplify OA grading with MRI for large-scale screening^10^. A pilot study demonstrated a potential correlation between structural phenotypes and OA progression^11^. Larger cohort studies with MRI assessment may further demonstrate the prognostic value of morphological phenotypes in predicting incident OA. ROAMES phenotypes are commonly seen in knees with OA; however, a large cohort study may corroborate the association between ROAMES phenotypes and incident OA in knees with pre-OA. Previous groups have circumvented issues of mass radiologic annotation by using artificial intelligence, which has provided high sensitivity and specificity in classifying knee structures in accordance with validated semi-quantitative grading scales, including anterior cruciate ligament, meniscus, and cartilage^12–14^.

Artificial intelligence may thus be applied to currently available large datasets of MRIs to associate morphological phenotypes with OA and future total knee replacement (TKR) surgery. Herein, our aim was to (1) build a fully automatic end-to-end deep learning model to stratify knees into pre-defined ROAMES phenotypes and (2) evaluate the prevalence and association of phenotypes with knee OA to better inform patient selection in clinical trials. Specifically, we investigated cross-sectional association between baseline morphological phenotypes and baseline structural OA and symptomatic OA. Among participants without baseline OA, we evaluated association of baseline phenotypes with 48-month structural OA and symptomatic OA. Lastly, we examined associations between phenotypes and undergoing TKR by 96 months from baseline.

## Materials and methods

### Study participants

We obtained the data from Osteoarthritis Initiative (OAI), which enrolled 4796 participants, aged 45 to 80 years, between 2005 and 2006 at four US centers. Participants in OAI had OA or were at high risk of developing OA in at least one knee at baseline. Participants at each site were assessed annually and evaluated information included questionnaires, physical examination, radiographic imaging, and MRI. Exclusion criteria included rheumatic or other inflammatory arthritis, contraindication to MRI, end-stage knee OA bilaterally, and inability to walk without assistance. National Institute of Arthritis and Musculoskeletal and Skin Disease approved the OAI study; the OAI was carried out in accordance with relevant guidelines and regulations (registered as “Osteoarthritis Initiative (OAI): A Knee Health Study”, NCT#00080171, on ClinicalTrials.gov). Participants provided informed consent at each study visit. The full trial protocol, eligibility criteria, and interventions have been previously documented^15–17^.

### Imaging

MRIs were collected using 3T scanners (Siemens Trio, Germany) on both right and left knees. From the OAI database, we accessed coronal intermediate-weighted two-dimensional turbo spin-echo (echo time/repetition time = 29 ms/3700 ms, field of view = 140 mm, matrix = 384 × 307, slice thickness = 3 mm, echo train length = 7, bandwidth = 352 Hz/pixel, excitations = 1, sections = 35) and two-dimensional sagittal intermediate-weighted fat-suppressed turbo spin-echo (echo time/repetition time = 30 ms/3200 ms, field of view = 160 mm, matrix = 448 × 313, slice thickness = 3 mm, echo train length = 5, bandwidth = 248 Hz/pixel, excitations = 1, sections = 37) sequences from all participants during all clinic visits. Five participants were excluded because they did not have both coronal and sagittal sequences, resulting in 4791 eligible study participants.

### MRI-based morphological phenotyping

ROAMES is a simplified MRI assessment metric for stratification of knees into morphological phenotypes potentially applicable to determine eligibility for DMOAD trials^10^. Subchondral bone phenotype is defined as knees with bone marrow edema in greater than 66% (MRI Osteoarthritis Knee Score^18^ (MOAKS) 3) of any of patellofemoral, medial tibiofemoral, or lateral tibiofemoral knee compartments. The meniscus/cartilage phenotype possesses knees with meniscus damage (MOAKS 6–8) on either the medial or lateral knee with ipsilateral cartilage damage (MOAKS 2.1, 2.2, 3.2, 3.3) and contralateral meniscal damage (MOAKS 2–8). Inflammatory phenotype is defined as knees with either inter-condylar synovitis or whole knee effusion with MOAKS grade 3, with at least a MOAKS 2 in the other respective feature. Hypertrophy phenotype consists of large osteophytes (MOAKS 3) and minimal cartilage damage (MOAKS 0–1) in any knee compartment.

A subset of the knee MR images from OAI were graded according to MOAKS as part of several previous studies and shared publicly, the first being the OA Biomarkers Consortium FNIH Project which studied 600 participants in a case-control study of OA incidence^19^. In 2017, MOAKS readings were also released for four other projects including case-control studies in 574 participants for studying incident lateral compartment OA^20^, in 613 participants for studying incident radiographic OA^21^, and the Pivotal OAI MR Imaging Analyses and a subcohort study of 850 participants with bilaterally normal knees at baseline^22^. Details of these five projects are publicly available (Supplemental File 1). The MOAKS grading was performed by a centralized group under the supervision of two musculoskeletal radiologists with more than nine years of training in semi-quantitative knee OA grading^17^. The radiologists were blinded to the clinical data and case-control status. A total of 2653 unique participants were imaged at either or both of two visits (baseline, 4 years), resulting in 4413 knee MRIs for grading in a total of 3117 unique knees. Baseline demographics for the participants were as follows: Women = 1574, Men = 1074, Age (mean[SD]) = 60.9 [9.0], BMI (mean[SD]) = 28.5 [4.8]), and the baseline Kellgren-Lawrence (KL) grades of the knees were KL0 = 1212, KL1 = 654, KL2 = 626, KL3 = 446, KL4 = 170.

Since ROAMES is a simplification of MOAKS, we used the radiologist MOAKS grades to directly assign ROAMES phenotypes of bone, meniscus/cartilage, inflammatory, and hypertrophy. This subset of OAI images with assigned ROAMES phenotypes was then used as ground truth for training neural network classifiers. The sample size of cases and controls from the ROAMES assigned OAI subset for bone, meniscus/cartilage, inflammatory, and hypertrophy were 532 and 3109, 101 and 3535, 50 and 1906, and 57 and 543, respectively. Every knee was not necessarily graded for each aspect of MOAKS. For example, some knees were graded for osteophytes, while others were not. Knees that did not have MOAKS grades necessary to determine presence or absence of a phenotype could not be used for training the particular neural network for that phenotype, which is why there are different sample sizes among the phenotypes. Knees graded with MOAKS grades to determine presence or absence of more than one phenotype subsequently were used for training each eligible phenotype. Thus, each classifier had knees that had non-exclusive phenotypes (i.e. training bone phenotype possessed cases that also had meniscus/cartilage phenotype). We trained four separate neural networks, so one knee could be a case in training one particular phenotype but may be a control in training a different phenotype classifier. Atrophy, defined as minimal osteophytes with severe cartilage damage, is the fifth ROAMES phenotype and was not included in this study due to low number of cases.

#### Model training for automated morphological phenotyping

Using the subset of the OAI with radiologist-assigned ROAMES phenotypes, we trained convolutional neural networks (CNNs) from coronal and sagittal knee MRIs to classify the four ROAMES phenotypes of bone, meniscus/cartilage, inflammatory, and hypertrophy. The radiologist-graded images were split into training (70%), validation (10%), and test sets (20%) for each CNN phenotype classifier, preserving the distributions of baseline demographics, radiographic severity, and pain severity. Images in these three splits for each phenotype classifier were from distinct, non-overlapping participants. The training set was used to train each of the CNNs with back propagation. Model performance after each training epoch was evaluated over the validation set. Test set was blind to the model until after training to serve as final metric of performance.

The CNNs utilized MRNet neural network architecture, which utilizes each slice of the concatenated coronal and sagittal views as input into an ImageNet pre-trained AlexNet for feature extraction^23^. The features from each slice were then pooled and input into a fully connected layer to produce a final binary classification probability assessing the presence or absence of phenotype. We trained one CNN for each phenotype over 80 training epochs with early stopping with following parameters: Adam optimizer, learning rates of 5 × 10^–5^ (bone CNN) and 1 × 10^–5^ (meniscus/cartilage, inflammatory, and hypertrophy CNNs), empirically-weighted cross-entropy loss to account for class imbalances, and batch size of 1. These model configurations were selected through several iterations of empirical parameter selection based on previously solving similar classification tasks^12,24,25^. Training set augmentation consisted of random two-dimensional translations, rotations, and zooming. We then performed a systematic hyperparameter tuning of these CNNs with a grid search of differing architectures (AlexNet, ResNet, DenseNet), learning rates (1E-4, 1E-5, 1E-6), weight decays (None, 0.01), and dropout rates (0.1, 0.3, 0.5). The highest performing phenotype models from the grid search were compared to the empirically tuned CNNs. McNemar’s test was used to compare classification performance on the validation set to determine statistically significant differences between the phenotype classifiers. The higher performing CNN was used to infer on the test set and entire OAI. All CNNs were developed in Pytorch (Facebook, Menlo Park, CA), and computations were performed on NVIDIA (Santa Clara, CA) GeForce GTX Titan X graphics processing units.

#### Model inference for automated morphological phenotyping of entire OAI dataset

To investigate the associations between morphological phenotypes and knee OA outcomes, the trained CNNs were then utilized to predict morphological phenotypes for the entire cohort’s bilateral knee images; specifically, we studied 4971 baseline patients over 8 study time points and obtained images from both knees at each visit. This resulted in a total of 45,300 MRI exams that were analyzed with both coronal and sagittal MRI views. To understand the prognosis effects of each phenotype, we chose one knee per participant and allowed maximum one phenotype per each participant, excluding samples fulfilling more than one phenotype. We chose the knee with greater radiographic severity or a random knee if severity was equal. The predicted morphological phenotypes served as the primary independent variables.

### Statistical analysis

We compared ROAMES predictions on the test set images from the CNNs with the corresponding ground truth radiologist assigned ROAMES phenotypes, which served to evaluate phenotype classification metrics of the CNNs. Performance measures included area under the curve (AUC), accuracy, sensitivity, and specificity. In these metrics, the true value was the radiologist phenotype and the predicted value was the model phenotype prediction. Standard errors were calculated using bootstrapping principle. One-way ANOVA tests compared training, validation, and test set demographics, radiographic scores, and pain scores.

Baseline characteristic differences were assessed between participants without phenotype and participants with each of the four morphological phenotypes using Kruskal–Wallis test for continuous variables or Chi-square test for categorial variables. Benjamini–Hochberg method was used for P-value adjustment as needed.

The primary outcome was structural OA and symptomatic OA. Structural OA was defined as KL radiographic grading scheme greater than or equal to 2 (presence of definite osteophyte)^26^. Symptomatic OA was defined as the presence of pain, aching, or stiffness in knee joint for most days lasting at least one month in past 12 months^27^. We investigated the association between baseline phenotypes and concurrent structural and symptomatic OA among all participants using logistic regression. In a longitudinal model, we evaluated the association of baseline phenotypes with incidence of structural OA and symptomatic OA at 48 months among participants without OA at baseline using mixed effects logistic regression analyses to account for multiple observation by participants. We additionally assessed the association between phenotypes and undergoing primary TKR after baseline and prior to the 96-month visit using logistic regression. Both cross-sectional and longitudinal model were adjusted for baseline characteristics, including age, sex, race, and body mass index (BMI), by adding these variables as predictors to the regressions. We built an additional TKR logistic regression model adjusted for symptomatic OA and KL by similarly adding baseline symptomatic OA and KL grade as predictors in the model. The definitory time point of phenotype characterization was baseline.

Two-tailed *P*-values less than 0.05 were considered statistically significant. Statistical analyses were performed in R environment for statistical computing and important packages included lme4 and car^28^.

## Results

### Automated morphological phenotyping performance

**Figure 1:**
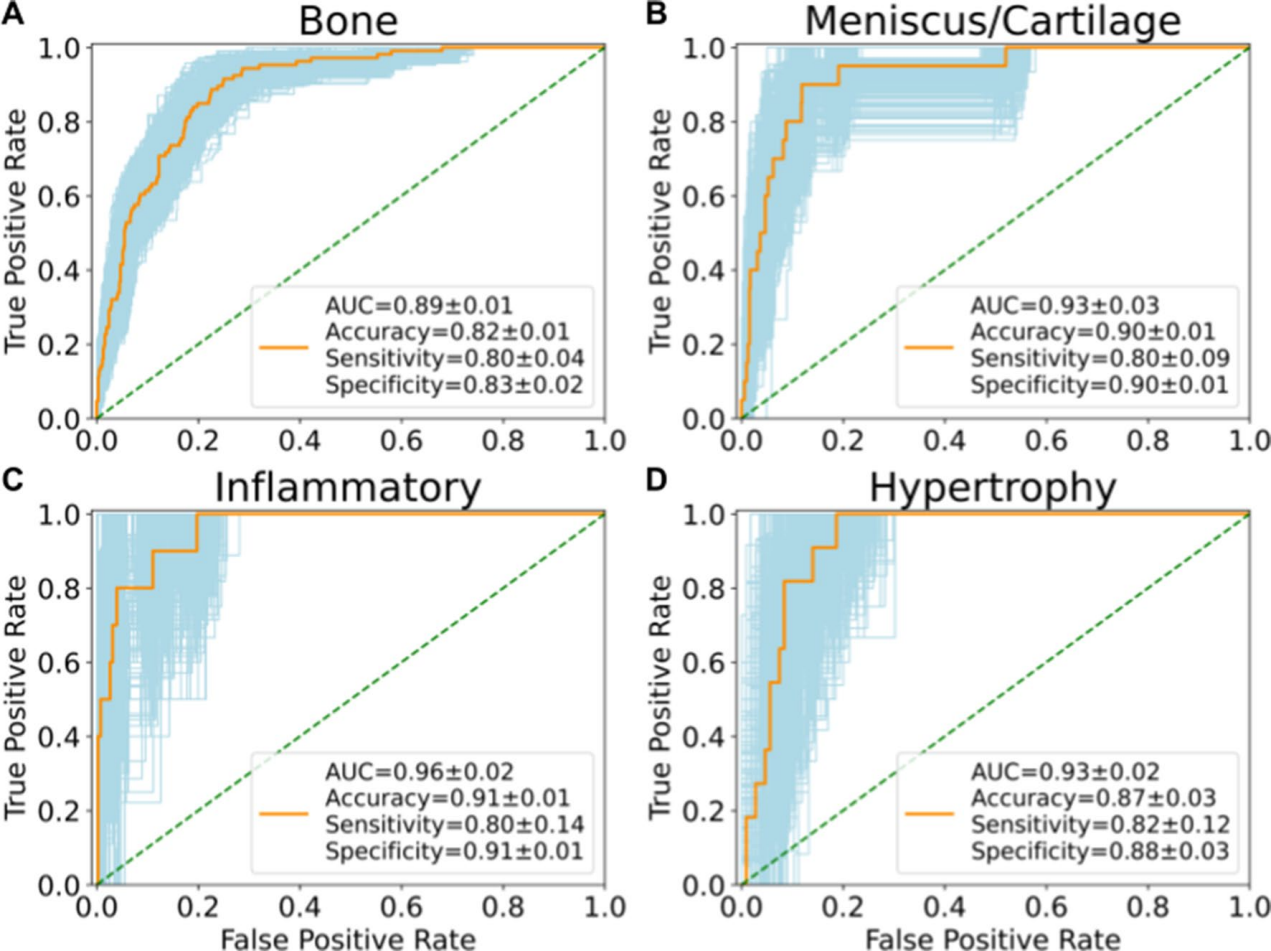
Receiver operating characteristic curves for knees from testing set (part of OAI subset graded by radiologists) with area under curve (AUC), accuracy, sensitivity, and specificity of the neural network phenotype classifiers. The AUC compared the predicted ROAMES phenotype from model prediction with the true phenotype assigned by the radiologists for each knee in the testing set. Metrics reported in mean ± standard deviation. Receiver operating characteristic curves produced using Python package Scikit-learn^29^.

There were no statistically significant differences between participants in the training, validation, and test sets regarding demographics, radiographic scores, and pain scores (Supplemental Table 1). The highest performing CNN from grid search for bone (model: AlexNet, learning rate: 1E-5, weight decay: 0, dropout: 0.3) and meniscus/cartilage (model: AlexNet, learning rate: 1E-4, weight decay: 0.01, dropout: 0.1) had significantly greater classification performance on validation set compared to their respective empirically tuned CNNs (*p* = 0.03 and *p* < 0.001, respectively). The optimally performing models from grid search for inflammatory (model: AlexNet, learning rate: 1E-5, weight decay: 0.01, dropout: 0.3) and hypertrophy (model: AlexNet, learning rate: 1E-5, weight decay: 0, dropout: 0.3) performed similarly to their respective empirically tuned CNNs (*p* = 0.34 and *p* = 0.99, respectively). The grid search CNNs were subsequently used for inference on test set and entire OAI. The AUCs of bone, meniscus/cartilage, inflammatory, and hypertrophy CNN classifiers for test set classification were 0.89 ± 0.01, 0.93 ± 0.03, 0.96 ± 0.02, and 0.93 ± 0.02, respectively (Fig. 1). The overall accuracy of each classifier was 82 ± 1% (598/727), 90 ± 1% (652/726), 91 ± 1% (354/390), and 87 ± 3% (103/118), respectively. Sensitivities of the neural networks were 80 ± 4% (80/106), 80 ± 9% (16/20), 80 ± 14% (8/10), and 82 ± 12% (9/11), respectively; specificities were 83 ± 2% (513/621), 90 ± 1% (636/706), 91 ± 1% (346/380), and 88 ± 3% (94/107), respectively.

### Morphological phenotyping of entire OAI dataset

**Figure 2:**
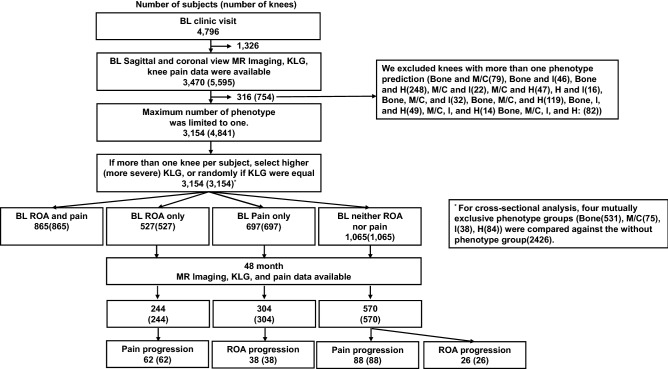
Flow chart of subject selection for phenotype analyses. BL: baseline, KLG: Kellgren Lawrence grading, ROA: radiographic OA, M/C: meniscus/cartilage, I: Inflammatory, H: hypertrophy.

A total of 754 knees that fulfilled more than one phenotype criteria were excluded. The final cohort included 3154 unique knees (Fig. 2). At baseline, the cohort contained 531 (16.8%) bone phenotype, 75 (2.4%) meniscus/cartilage phenotype, 38 (1.2%) inflammatory phenotype, 84 (2.6%) hypertrophy phenotype, and 2426 (76.9%) without phenotype (Table 1). Those in all four phenotype groups significantly differed from participants in no phenotype group in baseline KL grades and Knee Injury and Osteoarthritis Outcome Score (KOOS) pain score. The distributions of age and sex were similar in all phenotype groups, except meniscus/cartilage, compared to no phenotype group. Meniscus/cartilage phenotype group were older and consisted of more males relative to no phenotype group. BMI differed significantly in bone and hypertrophy phenotype groups in comparison to no phenotype group.

**Table 1:** Baseline characteristics for the analytic sample by phenotype group.

Characteristic*	Phenotypes				
	No Phenotype N = 2426 (76.9%)	Bone N = 531 (16.8%)	Meniscus/Cartilage N = 75 (2.4%)	Inflammatory N = 38 (1.2%)	Hypertrophy N = 84 (2.6%)
Age, years	60 (53–69)	59 (53–68)	67 (60–72)	63 (57–70.8)	64 (54.8–70)
*P* value	–	0.25	< 0.001	0.10	0.10
Sex Female, No. (%)	1383 (57.0)	326 (61.4)	29 (38.7)	18 (47.4)	26 (66.7)
*P* value	–	0.13	< 0.001	0.31	0.13
BMI kg /m^**2**^	27.7 (24.6–31.1)	28.8 (26.0–32.2)	28.7 (26.1–32)	27.6 (25.8–29.5)	32.8 (29.9–35.4)
*P* value	–	< 0.001	0.07	0.84	< 0.001
**Baseline KL grade, No. (%)**					
0 and 1	1544 (63.6)	201 (37.9)	5 (6.7)	8 (21.1)	4 (4.8)
2	624 (25.7)	204 (38.4)	21(28.0)	14(36.8)	41 (48.8)
3	242 (10.0)	100 (18.8)	38(50.7)	15(39.5)	35 (41.7)
4	16 (0.7)	26 (4.9)	11(14.7)	1(2.6)	4 (4.8)
*P* value	–	< 0.001	< 0.001	< 0.001	< 0.001
Baseline KOOS pain score	97.2 (78.2–100)	83.3 (66.7–6.9)	77.8 (68.1–95.8)	69.1 (50.7–85.7)	75.0 (59.7–88.9)
*P* value	–	< 0.001	< 0.001	< 0.001	< 0.001
Baseline radiographic OA, No. (%)	882 (36.4)	330 (62.1)	70 (93.3)	30 (78.9)	80 (95.2)
*P* value	–	< 0.001	< 0.001	< 0.001	< 0.001
Baseline symptomatic OA, No. (%)	1047 (43.2)	373 (70.2)	52 (69.3)	30 (78.9)	60 (71.4)
*P* value	–	< 0.001	< 0.001	< 0.001	< 0.001

*Median (interquartile range) or percentage.

-: not applicable, BMI: body mass index, KL: Kellgren-Lawrence, KOOS: Knee Injury and Osteoarthritis Outcome Score.

### Cross-sectional associations between morphological phenotype and structural and symptomatic OA

The proportions of participants who had structural OA and symptomatic OA at baseline were 44.1% and 49.8%, respectively. In adjusted logistic regression analyses, participants at baseline in bone (OR 2.76; 95% CI, 2.26–3.37), meniscus/cartilage (OR 22.9; 95% CI, 9.13–57.6), inflammatory (OR 6.40; 95% CI, 2.89–14.1), and hypertrophy (OR 24.7; 95% CI, 8.94–68.3) phenotype groups had significantly more structural OA than those in no phenotype group (Table 2). Symptomatic OA was significantly higher among participants in bone (OR 3.02; 95% CI, 2.46–3.72), meniscus/cartilage (OR 3.53; 95% CI, 2.13–5.86), inflammatory (OR 5.82; 95% CI, 2.63–12.9), and hypertrophy (OR 2.96; 95% CI, 1.81–4.85) phenotype groups compared to those in no phenotype group.

**Table 2:** Logistic regression models of cross-sectional structural and symptomatic OA (N = 3154). Both models were adjusted for age, sex, and BMI by adding these variables as predictors in the regression models.

Variable	Structural OA Odds ratio (95% CI)	Symptomatic OA Odds ratio (95% CI)
Baseline OA, N (%)	1392 (44.1)	1562 (49.8)
**Phenotypes**		
None	1 [reference]	1 [reference]
Bone	2.76 (2.26–3.37)*	3.02 (2.46–3.72)*
Meniscus/Cartilage	22.9 (9.13–57.6)*	3.53 (2.13–5.86)*
Inflammatory	6.40 (2.89–14.1)*	5.82 (2.63–12.9)*
Hypertrophy	24.7 (8.94–68.3)*	2.96 (1.81–4.85)*
Age	1.04 (1.03–1.05)*	0.96 (0.95–0.97)*
**Sex**		
Male	1 [reference]	1 [reference]
Female	1.09 (0.93–1.27)	1.07 (0.92–1.24)
BMI	1.10 (1.08–1.12)*	1.04 (1.03–1.06)*

“-”: not applicable, OA: osteoarthritis, CI: confidence interval, BMI: body mass index.

*: Statistically significant at *P* value < 0.05.

### Longitudinal associations between morphological phenotype and OA outcomes

We performed longitudinal analyses in only those subjects who had no OA at baseline and had 48 months follow up assessment. We analyzed 874 and 814 subjects at baseline for structural OA and symptomatic OA, respectively. Among the respective groups, 64 subjects developed structural OA and 150 subjects developed symptomatic OA at 48 month follow up. We only considered bone phenotype for structural OA because the number of baseline knees with meniscus/cartilage, inflammatory, and hypertrophy phenotypes who developed structural OA at 48 months were 1, 3, and 0, respectively. Participants in bone phenotype (OR 2.99; 95% CI, 1.59–5.62) had significantly higher adjusted odds of developing OA at 48 months compared to no phenotype group (Table 3). For symptomatic OA, we excluded inflammatory phenotype (n = 6). Among those without symptomatic OA at baseline, hypertrophy (OR 5.80; 95% CI, 1.82–18.5) phenotype was associated with a significantly higher adjusted odds of developing symptomatic OA at 48 months (Table 4).

**Table 3:** Association between phenotypes and incidence of structural OA within 48 months from baseline among participants without OA at baseline (n = 874). Model adjusted for age, sex, and BMI.

Variable	Number of samples (number of progression)	Odds ratio (95% CI)
**Phenotypes**		
None	769 (44)	1[Reference]
Bone	94 (16)	2.99 (1.59–5.62)*
Meniscus/Cartilage	3 (1)	–
Inflammatory	7 (3)	–
Hypertrophy	1 (0)	–
Age		1.01 (0.98–1.03)
**Sex**		
Male		1[Reference]
Female		1.11 (0.65–1.90)
BMI		1.09 (1.03–1.16)*

**Table 4:** Association between phenotypes and incidence of symptomatic OA within 48 months from baseline among participants without OA at baseline (n = 814). Model adjusted for age, sex, and BMI.

Variable	Number of samples (number of progression)	Odds ratio (95% CI)
**Phenotypes**		
None	716 (121)	1[Reference]
Bone	69 (16)	1.41 (0.77–2.58)
Meniscus/Cartilage	10 (4)	3.29 (0.89–12.1)
Inflammatory	6 (1)	–
Hypertrophy	13 (8)	5.80 (1.82–18.5)*
Age		1.01 (0.99–1.03)
**Sex**		
Male		1[Reference]
Female		1.46 (1.01–2.12)*
BMI		1.08 (1.04–1.13)*

A total of 147 (4.66%) subjects underwent TKR in our analytic sample (n = 3154). In logistic regression analysis unadjusted for baseline KL grade, all four phenotypes were associated with significantly increased odds of undergoing TKR within 96 months (Table 5). After adjustment for baseline KL grades and presence of pain, aching, and stiffness in knee joint at baseline, bone (OR 2.11; 95% CI, 1.39–3.19), inflammatory (OR 5.69; 95% CI, 2.43–13.4), and hypertrophy (OR 2.65; 95% CI, 1.35–5.19) phenotypes were associated with significantly increased adjusted odds of undergoing TKR within 96 months.

**Table 5:** Association between phenotypes and undergoing primary TKR, with and without adjustment for symptomatic OA and KL grade, after baseline and prior to the 96-month visit (n = 3154). The adjustment refers to adding baseline symptomatic OA and KL grade as additional predictors in the logistic regression model. Both models adjusted for age, sex, and BMI.

Variable	Number of samples (number of cases underwent TKR in 96 months)	TKR cases Odds ratio (95% CI)	TKR cases—with adjustment Odds ratio (95% CI)
**Phenotypes**			
None	2426(62)	1[Reference]	1[Reference]
Bone	531(53)	4.07 (2.77–5.97)*	2.11 (1.39–3.19)*
Meniscus/cartilage	75 (7)	3.73 (1.63 –8.54)*	0.88 (0.37–2.13)
Inflammatory	38(10)	13.6 (6.31–29.5)*	5.69 (2.43–13.4)*
Hypertrophy	84(15)	6.67 (3.31–12.7)*	2.65 (1.35–5.19)*
Age		1.02 (1.00–1.04)*	1.02 (0.98–1.02)
**Sex**			
Male		1[Reference]	1[Reference]
Female		1.43 (0.99–2.05)	1.71 (1.16–2.50)*
BMI		1.03 (0.99–1.07)	0.99 (0.96–1.03)
Baseline symptomatic OA		–	1.65 (1.38–2.48)*
**Baseline KL grade**			
0–1		–	1[Reference]
2		–	5.47 (2.89–10.4)*
3		–	17.6 (9.24–33.6)*
4		–	52.8 (22.9–122)*

“-”, not applicable; OA, Osteoarthritis; CI, confidence interval; BMI, body mass index; TKR, total knee replacement; KL, Kellgren-Lawrence.

*Statistically significant at *P* value < 0.05.

## Discussion

We built an end-to-end deep learning model to rapidly stratify knees into morphological phenotypes using a large, longitudinal cohort. We examined associations of phenotypes with odds of concurrent OA, obtaining OA within 48 months from baseline, and receiving TKR surgery within 96 months from baseline. All phenotypes, particularly meniscus/cartilage and hypertrophy, were associated with concurrent structural OA. Additionally, all phenotypes increased odds of concurrent symptomatic OA. Among knees with no baseline OA, bone phenotype and hypertrophy phenotype each respectively increased odds of incident structural OA and symptomatic OA in 48 months. All phenotypes except meniscus/cartilage increased odds of undergoing TKR within 96 months after adjustment for baseline KOOS score and KL grade. Identifying phenotypes of knee OA may aid in stratifying patients for clinical trials and guide development of targeted interventions to prevent disease progression^1,30^.

Roemer et al. conducted a study associating ROAMES phenotypes with OA in a cohort of 485 knee MRIs with a priori-defined outcomes from FNIH^11^. They reported knees, with KL grades 2 and 3, possessing bone phenotype at baseline had highest odds of structural OA at either 24, 36, or 48 months (OR 1.87; 95% CI, 1.18–2.97). Neither bone, meniscus/cartilage, nor inflammatory phenotypes increased odds of pain progression over the same study period. Our study similarly determined bone phenotype to increase incident structural OA and that bone and meniscus/cartilage did not increase odds of incident symptomatic OA. However, hypertrophy phenotype did increase odds of symptomatic OA in our study. Roemer et al. did not report hypertrophy phenotype due to sample size constraints, and there is little literature evaluating hypertrophy phenotype in relation to incident OA. Compared to Roemer et al., we did not exclude knees based on KL grade, whereas Roemer et al. excluded all knees with KL less than 2. They also defined structural progression as a decrease in minimal joint space width of at least 0.7 mm in the medial tibiofemoral joint. The authors also utilized Western Ontario and McMaster Universities Osteoarthritis Index to assess symptomatic progression, whereas our study examined minimally detectable change in KOOS. Finally, both studies had different sample sizes and study lengths.

Cross-sectional analysis of baseline characteristics demonstrated a significant proportion of radiographic OA among knees with any phenotype, the highest proportion of which appearing in meniscus/cartilage and hypertrophy. These two phenotypes most overlap with criteria for radiographic OA, defined as definite evidence of osteophytes and joint space narrowing. Knees fulfilling criteria for either phenotype but not structural OA may be reflective of decreased sensitivity of x-ray in detecting osteophytes and cartilage degeneration relative to MRI^31,32^. Limited specificity of the CNNs may also contribute to the discrepancy. Although these two phenotypes generated highest odds of concurrent structural OA, inflammatory phenotype was most associated with concurrent symptomatic OA. Increased odds of effusion-synovitis observed two years prior to incident radiographic OA has been documented; moreover, weight and sex of the subjects can further augment this odds ratio^33^. Our logistic regression model similarly found BMI as predictive of concurrent OA, though we did not find a sex-dependent relationship.

The majority of subjects without OA at baseline did not have a phenotype. Despite low prevalence, bone phenotype significantly increased odds of incident structural OA at 48 months. It is difficult to put this finding into perspective as odds ratios could not be computed for any other phenotype given their limited sample sizes. Nonetheless, changes in subchondral bone have been reported as biomarkers of incident OA^34^. Specifically, morphological maps of bone shape analyzed by artificial intelligence were found to be predictive of incident OA. Damage to subchondral bone has been hypothesized to be a precursor to subsequent cartilage deterioration and a mediator of early resorptive phases in OA^35–37^. Notably, we did not find an association between bone phenotype and incident symptomatic OA at 48 months, but rather there was a relationship with hypertrophy phenotype. Although inflammatory phenotype possessed highest odds of symptomatic OA in cross-sectional analysis, the sample size was too limited to discern conclusions regarding longitudinal effects on symptomatic OA.

In longitudinal analysis of incident TKR in 96 months, KL grade portended highest odds when added to the regression. With this adjustment, all phenotypes except meniscus/cartilage demonstrated increased odds of TKR, suggesting incorporation of phenotypes can further stratify risk among subjects with similar KL scores. Of the phenotypes, inflammatory increased odds of incident TKR more than bone or hypertrophy. A prior study investigating predictive factor of MRI for incident TKR demonstrated tibiofemoral joint cartilage and bone, as well as medial and lateral menisci, were significant structures for accurate predictions by neural networks^38^. The study did not evaluate potential contribution from lesioned synovium or effusions, which should be explored in future works. Longitudinal changes in physical activity and pain have been reported to be unaffected by baseline cartilage damage^39^, which may corroborate our findings that meniscus/cartilage phenotype did not independently increase odds of incident TKR.

Despite relatively satisfactory performance metrics from the CNNs, methods using deep learning are limited. Artificial intelligence may serve as a valuable aid for clinicians and researchers with high workload or limited expertise, but detailed evaluation of relevant pathology by radiologists is inevitably necessary for accurate staging and diagnosis. Other limitations include use of MRI instead of arthroscopy as reference. The grades used for model training are dependent on subjective assessment by a radiologist, and our model can only perform as good as the MRI standard used in training. Moreover, OA is multifactorial, and future model building should include genetic, biochemical, and post-traumatic data. We also did not exclude posterior medial meniscus root tears, osteonecrosis, or malignancies which are typically exclusion criteria in DMOAD trials. In future work, we aim to develop CNNs to automatically detect these pathologies from large study cohorts. Inferring on samples from other studies is particularly important to demonstrate external validity of the CNNs, given our study results were not validated on an external cohort such as the Multicenter Osteoarthritis Study^40^. Another aim is to build a single multi-label classifier to compare with the current approach of a separate classifier for each phenotype. Multi-label models offer generalizability, interpretability, and less overfitting; however, they are limited by the label with the lowest sample size, which in our case was hypertrophy phenotype.

In conclusion, our study underscores the prognostic value of morphological phenotypes for characterizing progression of knee OA. These findings hold implications for improving understanding of OA pathogenesis, which may guide inclusion criteria of DMOAD trials towards MRI-based structural phenotypes. This may improve effectiveness of DMOADs by using individual knee phenotypes to offer patient-specific treatment. Future research can survey individual DMOAD trials to analyze whether specific subgroups of structural phenotypes received increased therapeutic benefits.

## Supplementary Information

Supplementary Table 1.

Supplementary Information.
