# Supplementary material for: Deep learning for large scale MRI-based morphological phenotyping of osteoarthritis
Source: Sci Rep. 2021 May 25;11:10915. doi: 10.1038/s41598-021-90292-6 (PMC8149826; doi:10.1038/s41598-021-90292-6)
Supplement: Supplementary file 1 — Supplementary Table 1. [file 41598_2021_90292_MOESM1_ESM.docx]

**Title**: Deep Learning for Large Scale MRI-Based Morphological Phenotyping of Osteoarthritis

**Authors**: Nikan K. Namiri, BS,^1^ Jinhee Lee, MS, ^1^ Bruno Astuto, PhD, ^1^ Felix Liu, MS, ^1^ Rutwik Shah, MD, ^1^ Sharmila Majumdar, PhD,^1^ Valentina Pedoia, PhD^1^

**Affiliations**: ^1^Department of Radiology and Biomedical Imaging and Center for Intelligent Imaging, University of California, San Francisco, San Francisco, CA, USA

**Corresponding Author**: Valentina Pedoia, PhD; [valentina.pedoia@ucsf.edu](mailto:valentina.pedoia@ucsf.edu); 415-476-6830; Department of Radiology and Biomedical Imaging, University of California, San Francisco, 1700 Fourth St, Suite 201, QB3 Building, San Francisco, CA 94107

**Supplementary Material**

**Supplemental Table 1.** Distribution of training, validation, and test sets for the neural network classifiers. Cases are knees fulfilling the ROAMES phenotype; controls are knees without the respective phenotype. Control phenotypes were used as a case in other phenotype classifier(s) if they met grading criteria for other phenotypes. These knees were derived from the radiologist-graded subset of the OAI graded by radiologists, which included 2,653 unique participants were imaged at either or both of two visits (baseline, 4 years), resulting in 4,413 knee MRIs for grading in a total of 3,117 unique knees. Baseline demographics for the participants were as follows: Women=1574, Men=1,074, Age (mean[SD]) = 60.9 [9.0], BMI (mean[SD]) = 28.5 [4.8]), and the baseline KL grades of the knees were KL0 = 1212, KL1 = 654, KL2=626, KL3=446, KL4=170.

|  | Bone | |  |  | | |  | Meniscus/Cartilage | | |  | | Inflammatory | | |  | | |  | Hypertrophy | | | |  | |  | |
| --- | --- | --- | --- | --- | --- | --- | --- | --- | --- | --- | --- | --- | --- | --- | --- | --- | --- | --- | --- | --- | --- | --- | --- | --- | --- | --- | --- |
|  | | Training | Validation | | Test | P value | | Training | Validation | Test | | P value | | Training | Validation | | Test | P value | | | Training | Validation | Test | | P value | |  |
| Case MRIs | | 372 | 54 | | 106 |  | | 71 | 10 | 20 | |  | | 35 | 5 | | 10 |  | | | 40 | 6 | 11 | |  | |  |
| Control MRIs | | 2175 | 313 | | 621 |  | | 2474 | 355 | 706 | |  | | 1335 | 191 | | 380 |  | | | 381 | 55 | 107 | |  | |  |
| Gender (W), n (%) | | 1543 (61) | 236 (64) | | 435 (60) | 0.33 | | 1517 (60) | 234 (64) | 458 (63) | | 0.09 | | 849 (62) | 124 (63) | | 249 (64) | 0.77 | | | 248 (59) | 38 (62) | 67 (57) | | 0.78 | |  |
| Age, mean (SD) | | 63 (9) | 62 (9) | | 62 (9) | 0.13 | | 62 (9) | 63 (9) | 62 (9) | | 0.84 | | 62 (9) | 62 (9) | | 62 (9) | 0.60 | | | 61 (9) | 61 (9) | 63 (9) | | 0.29 | |  |
| BMI, mean (SD) | | 29 (5) | 29 (5) | | 28 (5) | 0.19 | | 29 (5) | 29 (5) | 28 (5) | | 0.18 | | 30 (5) | 29 (5) | | 29 (5) | 0.16 | | | 31 (5) | 30 (5) | 31 (5) | | 0.55 | |  |
| KL Grade, n(%) | |  |  | |  | 0.90 | |  |  |  | | 0.49 | |  |  | |  | 0.80 | | |  |  |  | | 0.48 | |  |
| 0 | | 946 (37) | 142 (39) | | 254 (35) |  | | 959 (38) | 125 (34) | 256 (36) | |  | | 226 (17) | 37 (26) | | 61 (16) |  | | | 0 (0) | 0 (0) | 1 (1) | |  | |  |
| 1 | | 535 (21) | 63 (17) | | 171 (24) |  | | 525 (21) | 90 (25) | 153 (21) | |  | | 365 (27) | 42 (21) | | 106 (27) |  | | | 50 (12) | 5 (8) | 20 (17) | |  | |  |
| 2 | | 518 (21) | 76 (21) | | 151 (21) |  | | 524 (21) | 64 (18) | 156 (22) | |  | | 336 (25) | 47 (24) | | 102 (26) |  | | | 216 (51) | 35 (57) | 55 (47) | |  | |  |
| 3 | | 384 (15) | 58 (16) | | 100 (14) |  | | 368 (15) | 66 (18) | 106 (15) | |  | | 273 (20) | 50 (26) | | 94 (24) |  | | | 155 (37) | 21 (34) | 42 (36) | |  | |  |
| 4 | | 143 (6) | 24 (7) | | 43 (6) |  | | 146 (6) | 20 (5) | 44 (6) | |  | | 152 (11) | 20 (10) | | 27 (7) |  | | | 0 (0) | 0 (0) | 0 (0) | |  | |  |
| KOOS, mean (SD) | | 85 (17) | 84 (18) | | 86 (16) | 0.36 | | 85 (17) | 84 (19) | 84 (18) | | 0.09 | | 82 (18) | 82 (17) | | 80 (19) | 0.10 | | | 84 (16) | 83 (17) | 84 (16) | | 0.84 | |  |
| WOMAC, mean (SD) | | 11 (15) | 12 (15) | | 11 (14) | 0.54 | | 11 (14) | 12 (15) | 12 (15) | | 0.31 | | 14 (16) | 14 (15) | | 15 (16) | 0.47 | | | 13 (15) | 13 (16) | 13 (14) | | 0.91 | |  |

MRIs: Magnetic Resonance Images, SD: standard deviation, BMI: body mass index, KL: Kellgren-Lawrence, KOOS: Knee Injury and Osteoarthritis Outcome Score, WOMAC: Western Ontario and McMaster Universities Osteoarthritis Index
