# Supplementary material for: Deep learning for large scale MRI-based morphological phenotyping of osteoarthritis
Source: Sci Rep. 2021 May 25;11:10915. doi: 10.1038/s41598-021-90292-6 (PMC8149826; doi:10.1038/s41598-021-90292-6)
Supplement: Supplementary file 2 — Supplementary Information. [file 41598_2021_90292_MOESM2_ESM.pdf]

Semi-Quantitative Scoring of Knee MRI using MOAKS

1. Overview 2
1.1 SAS dataset 2
1.2 Contents of dataset 2
1.3 Condition 2
1.4 Variables and reading methods 2
1.5 MOAKS Anatomical Locations for Scoring 3
1.5.1 Locations for Cartilage Morphology and Bone Marrow Lesion Scoring 3
1.5.2 Locations for Scoring Osteophytes 4
1.5.3 Locations for Scoring Meniscal Damage 4
1.5.4 Locations for scoring of Synovitis and Effusion 4
1.6 MOAKS Variables and grades for scoring of OA related changes in structure 4
1.6.1 Cartilage Scores 5
1.6.2 BML Scores 5
1.6.3 Osteophyte Scoring 5
1.6.4 Meniscus Scoring 6
1.7 Compartment-specific variables, predictors and outcomes 6
1.7.1 Compartment-specific grouping of variables 6
1.7.2 Calculating predictors, longitudinal changes and outcomes 6
2. Methods specific to Project 22 8
2.1 Image type 8
2.2 Time points 8
2.3 Measurement methods 8
2.4 Variables 8
2.5 Sample 8
3. Methods specific to Project 30 9
3.1 Image type 9
3.2 Time points 9
3.3 Measurement methods 9
3.4 Variables 9
3.5 Sample 9
4. Methods specific to Project 61 10
4.1 Image type 10
4.2 Time points 10
4.3 Measurement methods 10
4.4 Variables 10
4.5 Sample 10
5. Methods specific to Project 63 11
5.1 Image type 11
5.2 Time points 11
5.3 Measurement methods 11
5.4 Variables 11
5.5 Sample 11
6. Methods specific to Project 65 12
6.1 Image type 12
6.2 Time points 12
6.3 Measurement methods 12
6.4 Variables 12
6.5 Sample 12
7. References 14

1. Overview

1.1 SAS dataset

Name: **kMRI_SQ_MOAKS_BICLxx** (xx identifies the time point)
Display label: **kMRI SQ MOAKS (BICL)**

1.2 Contents of dataset

This dataset contains centrally performed longitudinal semi-quantitative (SQ) readings of OA related structural changes from MRI of the knee performed at Boston Core Imaging Lab (http://www.bicl.org) by Drs. Ali Guermazi and Frank Roemer. The MRI exams were read using the MOAKS (MRI Osteoarthritis Knee Score) scoring method[1].

Each dataset contains the reading data for a single visit, or time point. The corresponding longitudinal data for other time points are in separate datasets. For example, the data from this vendor's reading of baseline MRIs are in a dataset ending in "00" (i.e., kMRI_SQ_MOAKS_BICL00), while the corresponding data from this vendor for the 12-month visit MRI readings are in a dataset with the same name but ending in "01" (i.e., kMRI_SQ_MOAKS_BICL01), and data for the 24-month visit MRI readings are in a dataset with the same name but ending in "03" (i.e., kMRI_SQ_MOAKS_BICL03). (See the "VisitPrefixDefinitions.pdf" document for a guide to visit numbering). To compare values of a variable across time points from a given project by this vendor, or to calculate change scores, users will need to merge the datasets for the various time points.

1.3 Condition

- Known data errors: none at present.
- Dataset strengths/weaknesses:
  - Data are expected for all participants included in a project sample. If expected data do not exist, SAS special missing values are assigned to denote why the data were not obtained.
  - The dataset contains one row of data (record) for a given knee which needs to be taken into account when merging it with other datasets. Please see the "*Overview and Description of Central Image Assessments*" document for more information on merging.

1.4 Variables and reading methods

A complete listing of the variables in these datasets can be found in the documentation provided with the dataset, including SAS variable names, descriptive variable labels and attributes.

Variables assessed for using MOAKS include:

- Scores for cartilage morphology (lesion size and depth) in 14 anatomical locations in the knee.
- Scores for the size and number of bone marrow lesions (BMLs) in 15 anatomical locations.
- Scores for osteophyte size in 12 anatomical locations.
- Scores for meniscal damage for anterior horn, body and posterior horn of both medial and lateral menisci, plus meniscal signal abnormalities, root tears, meniscal hypertrophy and extrusion.
- A score for synovitis at infra-patellar fat pad and one for synovitis/effusion in the whole knee.
- Scores for cruciate ligament teats (ACL and PCL) and extra articular features (e.g.: cysts, bursitis).

This publication[1] gives more details about the scoring methods used:

- *Hunter DJ et al. Evolution of semi-quantitative whole joint assessment of knee OA: MOAKS (MRI Osteoarthritis Knee Score). Osteoarthritis and Cartilage 2011; 19(8); 990-1002. PMID: 21645627 PMCID: PMC4058435* http://dx.doi.org/10.1016/j.joca.2011.05.004

MRI signal in the ilio-tibial band is described as part of MOAKS, but was not scored in any of the reading projects included in this dataset.

See later sections for any differences between projects in the way that reading methods may be different from what is described below.

The datasets provided only provide information about the raw MOAKS scores for each feature/anatomical location but sections 1.7 and 1.8 give some information and examples about how raw variables can be combined to give various kinds of information such as:

- Does a knee have any kind of meniscal tear at baseline?
- Does cartilage morphology in the medial tibio-femoral compartment worsen between 2 visits?
- Has BML score in any patello-femoral location worsened between baseline and follow up? (regardless of whether any patello-femoral locations have shown improvement of BML score)

1.5 MOAKS Anatomical Locations for Scoring

1.5.1 Locations for Cartilage Morphology and Bone Marrow Lesion Scoring

MOAKS scores cartilage morphology and bone marrow lesions in a large number of anatomical locations. Figure 1 shows the 3 MOAKS subregions of the lateral tibial plateau (A = anterior, C=central and P=posterior), along with the 2 subregions of the femoral condyle (C=central and P=posterior) which together make up the 5 subregions of the lateral tibio-femoral compartment. There are 5 similar anatomical locations on the medial side of the joint which make up the medial tibio-femoral compartment.

Figure 1. Showing the anterior (A), central (C) and posterior (P) subregions of the lateral femoral condyle and lateral tibial plateau used in WORMS. There are similar regions defined for the medial side of the knee.

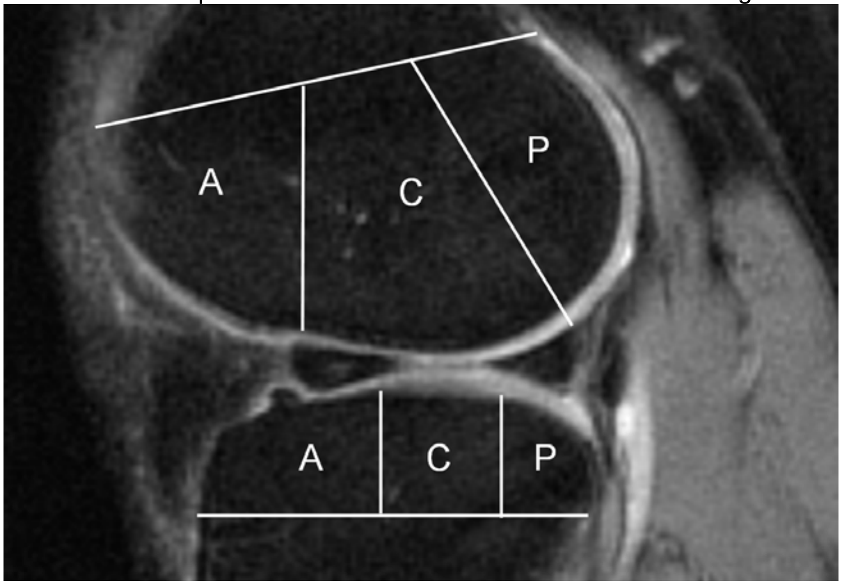

In MOAKS, the anterior (or trochlear portion) of the lateral femoral condyle (A) is considered part of the patello-femoral compartment since it articulates with the lateral facet of the patella. Similarly, the anterior of the medial femoral condyle, which articulates with the medial facet of the patella is part of the patello-femoral compartment. Therefore the patello femoral compartment comprises 4 anatomical subregions, 2 from the femur and 2 from the patella. The 4 patello-femoral compartment subregions, along with the 5 medial tibio-femoral compartment subregions and 5 lateral tibio-femoral compartment subregions comprise the 14 subregions used for scoring cartilage morphology. For bone marrow lesions (BMLs) there is an additional sub-spinous region (Figure 2) which is associated with the insertion of the cruciate ligaments rather than being associated with an articular surface. This feature is associated with the tibio-femoral joint, but is not assigned to either medial or lateral compartment of that joint. Figure 2 also shows the line used to differentiate medial and lateral sides of the femur.

Figure 2. Showing the lines delineating medial and lateral sides of the femur and tibia, along with the definition of the sub-spinous region (SS) used only for scoring bone marrow lesions in MOAKS.

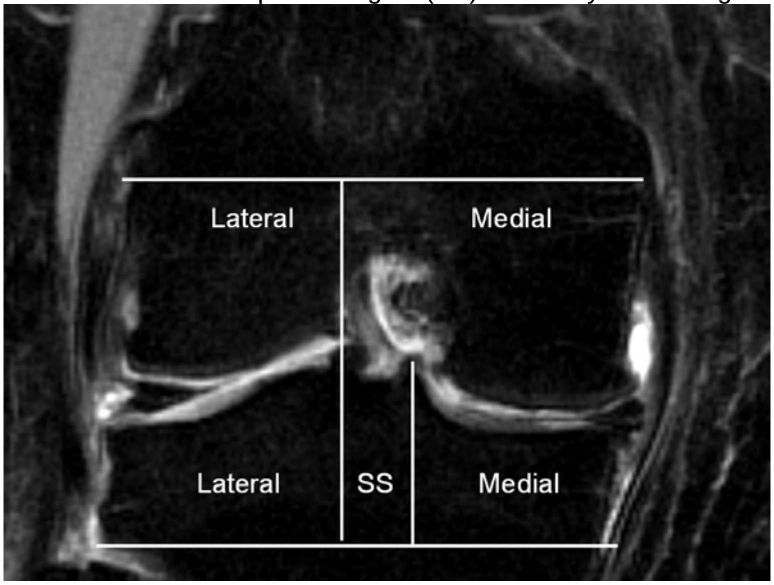

1.5.2 Locations for Scoring Osteophytes

Osteophytes in the patello-femoral joint were scored at 4 locations on the patella (superior, inferior, medial and lateral), as well as 2 locations (medial and lateral) on the anterior/trochlear portion of the femur. For the medial tibio-femoral joint osteophyte size on around the medial tibial plateau was scored as well as osteophyte size at two locations (central and posterior) on the medial femoral condyle. For the lateral tibio-femoral joint 3 similar locations were scored.

1.5.3 Locations for Scoring Meniscal Damage

For MOAKS, each meniscus (medial and lateral) was split into three subregions: anterior horn, meniscal body, and posterior horn. The presence and type of any meniscal tear was scored separately for each of those 6 subregions. Signal abnormalities that were not severe enough to be called tears were also recorded, as was the presence of any posterior root tears of either meniscus. Extrusion of the body of each meniscus (in the medio-lateral direction) was scored and anterior extrusion of the anterior horn was also scored.

1.5.4 Locations for scoring of Synovitis and Effusion

Synovitis was scored in the infra-patellar fat pad based on signal abnormalities in Hoffa's fat pad, and presence and size of synovial effusion was also scored. It is important to note that this effusion score can include both synovitis and effusion since it is impossible to differentiate the two using the non-enhanced MRI sequences used for this study.

1.6 MOAKS Variables and grades for scoring of OA related changes in structure

Variable names prefixed with V00 are for baseline values, V01 for 12-month visit values and V03 for 24-month visit values. Detailed descriptions, examples and definitions of the different score values each feature scored are given in the original publications for MOAKS[1]. Variables names tend to be made of 3 parts, (i) the visit prefix Vxx, (ii) a short abbreviation of the type of score (e.g.: "MCM" for MOAKS cartilage morphology, "MBMS" for MOAKS bone marrow lesion size, "MMT" for MOAKS meniscal tear, and (iii) a short abbreviation of the anatomical location (e.g.: "FMP" for femur medial posterior, "TLP" for tibia lateral posterior, both of which apply for cartilage and bone marrow lesions, or "MB" for medial body, which applies to meniscus).

A complete listing of the variables in these datasets can be found in the documentation provided with the dataset, including SAS variable names, descriptive variable labels and attributes.

The following sections describe details of some of the more important features.

1.6.1 Cartilage Scores

MOAKS scores the size of any cartilage lesions on a 4 point scale based on the percentage of the subregions that the lesion(s) affect. There is also a separate score for the percentage of the subregion that is affected by full thickness cartilage loss. Table 1 shows the thresholds used for each of these scores.

Table 1. Showing the values for MOAKS scores of cartilage morphology

Size of any cartilage loss (partial or full thickness) as a % of the surface area of the subregion	% full thickness cartilage loss in the subregion
0: none	0: none
1: < 10% of the surface area of the region	1: < 10% of the surface area of the region
2: 10-75% of the surface area of the region	2: 10-75% of the surface area of the region
3: >75% of the surface area of the region	3: >75% of the surface area of the region

In the dataset, these two scores are combined into a single number where the portion before the decimal point represents the score for the size of the lesion and the portion after the decimal point represents the score for the amount of full thickness cartilage loss.

So, for example, a value of 1.0 represents a small isolated cartilage lesion that covers less than 10% of the surface area of the subregion and there is no full thickness cartilage loss, and a grade 3.1 lesion represents a large lesion that covers more than 75% of the surface area of the subregion, but has only a small amount of full thickness cartilage loss covering less than 10% of the surface area of the subregion.

At follow-up visits only, a special value of 0.5 is used to record that although the score is the same as at the previous visit, a definite worsening has occurred. This is called a within-grade worsening. A special value of -0.5 is used to record when a within-grade improvement has occurred.

1.6.2 BML Scores

For each subregions analyzed, MOAKS has 3 separate scores, one for the % of the volume of the subregion that is affected by BML, one for the number of BMLs within the subregion and a 3rd score for the % of the lesion that is BML, as opposed to cyst. Table 2 shows size thresholds used for this. At follow-up visits only, a special value of 0.5 is used to record that although the score is the same as at the previous visit, a definite worsening has occurred. This is called a within-grade worsening. A special value of -0.5 is used to record when a within-grade improvement has occurred.

Table 2. Showing the scoring system for BMLs

Size of BML (including any associated cysts	Number of BMLs counted within the subregion	% of lesion that is BML (vs cyst)
0: none	0: no BMLs in subregion	0: none
1: < 33% of subregional volume	1: a single BML in the subregion	1: < 33%
2: 33-66% of subregional volume	2: a pair of BMLs in the subregion	2: 33-66%
3: >66% of subregional volume	etc	3: > 66%

1.6.3 Osteophyte Scoring

Osteophytes are scores on a 4 point scale: Grade 0 = none, Grade 1=small, Grade 2=medium, Grade 3=large. Examples of each grade are given in the original MOAKS publication1.

1.6.4 Meniscus Scoring

Abnormalities of the meniscus are scores as follows

0: normal meniscus
1: signal abnormality that is not severe enough to be considered a meniscal tear
2: radial tear
3: horizontal tear
4: vertical tear
5: complex tear
6: partial maceration
7: progressive partial maceration (only used for follow-up visit scores)
8: complete maceration

As well as recording those features, the presence of meniscal hypertrophy (a definite increase in the meniscal volume compared to normal) is recorded as well as the presence of any meniscal extrusion or meniscal cysts. Detailed definitions of these are given in the original MOAKS publication[1].

1.7 Compartment-specific variables, predictors and outcomes

1.7.1 Compartment-specific grouping of variables

The various anatomic locations used for cartilage morphology, bone marrow lesions and osteophytes can generally be grouped into one of the 3 compartments of the knee joint:

For cartilage morphology and bone marrow lesion scores, the following grouping can be used,

The medial tibio-femoral compartment comprises the 5 anatomical locations which are listed below along with the abbreviations used in the relevant variable names:

FMC – femoral condyle (medial) central region
FMP – femoral condyle (medial) posterior region
TMA – tibia (medial) anterior region
TMC – tibia (medial) central region
TMP – tibia (medial) posterior region

The lateral tibio-femoral compartment comprises the 5 anatomical locations which are listed below along with the abbreviations used in the relevant variable names:

FLC – femoral condyle (lateral) central region
FLP – femoral condyle (lateral) posterior region
TLA – tibia (lateral) anterior region
TLC – tibia (lateral) central region
TLP – tibia (lateral) posterior region

The patella-femoral compartment comprises the 4 anatomical locations which are listed below along with the abbreviations used in the relevant variable names:

FMA – femur (medial) anterior region
FLA – femur (lateral) anterior region
PM – patella medial facet
PL – patella lateral facet

1.7.2 Calculating predictors, longitudinal changes and outcomes

It is important to remember that the raw values for MOAKS variables for cartilage morphology and meniscal damage have to be thought of as categorical variables, and are scored in multiple locations.

This means that if users want to determine a compartment-specific predictor or outcome, multiple values/variables have to be considered.

So, for example, if the aim is to determine if the medial tibio-femoral compartment has any full thickness cartilage loss, the variables V00MCMTMC, V00MCMTMP, V00MCMTMA, V00MCMFMC, V00MCMFMP

have to be considered. If **ANY** of them is non-integer (take the value n.x where x is 1, 2 or 3), then that knee has full thickness cartilage loss in the medial tibio-femoral sub-compartment. If **ALL** of the 5 subregions have an integer valued score (0, 1, 2 or 3), then the compartment has no full thickness loss. It is important to note that if any of the 5 subregions have a missing score, it is still possible to determine that full thickness loss exists, but it is impossible to determine if it does not exist.

For cartilage morphology scores, a subregion can be considered as having worsened over time if any of the following occur between two visits:

(a) The size of the lesion has changed (i.e.: the integer portion of the score has gone from 0->1 , or from 1->2 or from 2->3)
(b) The amount of full thickness loss has increased (i.e.: the decimal potion of the raw score has increased (i.e.: 1.0->1.1, or 2.0->2.1, or 2.2, or 3.0->3.1, or 3.0->3.2, or 3.0->3.3, or 3.1->3.2, or 3.1->3.3, or 3.2->3.3
(c) A within-grade change of +0.5 has been recorded at the follow-up visit, although in some analyses, where a stricter definition of change is required, such within-grade changes may be considered as no-change and in those cases, the score at the previous visit needs examining.

For meniscal damage, scores of 0 or 1 are considered to not be tears. The remaining values are categorical and it is important to note that values of 2 “radial tear”, 3 “horizontal tear” and 4 “vertical tear” are not necessarily of increasing severity. A value of 5 “complex tear” or 8 “complete maceration” could be considered to be worse than values less than 5, but there is no reason to consider 6 “partial maceration” as worse than 5 “complex tear”, although 6 “partial maceration” is definitely less severe than 8 “complete maceration”.

These issues need careful consideration when using the raw values of variable to determine the status and severity of damage in compartments or knees, and even more careful consideration when determining longitudinal changes and calculation of outcomes of structural worsening in the knee.

2. Methods specific to Project 22

2.1 Image type
For this study, the sagittal and coronal IW TSE series, the sagittal 3D DESS WE, and the axial and coronal multiplanar reformats (MPRs) of the DESS series were used. For the MRI acquisition protocol, see the “MRI Manual” operation manual.

2.2 Time points
Baseline, 12-month, and 24-month visits.

2.3 Measurement methods
Prior to transferring images to BICL and the start of taking measurements, the MR images were blinded to the OAI Release ID. The images were assessed paired and with known chronological order under the supervision of Dr. Ali Guermazi. Cartilage morphology, BMLs, osteophytes, meniscal damage, ACL/PCL tear, synovitis and effusion and extra articular features such as cysts and bursitis were scored. MOAKS scoring as described in sections 1.4 – 1.7 was performed.

2.4 Variables
See the dataset documentation file *kMRI_SQ_MOAKS_BICLxx_Contents.pdf* in the compressed documentation file for a complete list of all the variables in the dataset, their SAS variable names, descriptive variable labels and attributes.

2.5 Sample
The knees studied for this project are from the OA Biomarkers Consortium FNIH Project. The data for these knees in these datasets (kMRI_SQ_MOAKS_BICLxx for Project 22) are duplicates of the data in the kMRI_FNIH_SQ_MOAKS_BICLxx datasets.

More details of this project are given in this publication[2]:

- *Collins JE et al. Semiquantitative Imaging Biomarkers of Knee Osteoarthritis Progression: Data From the Foundation for the National Institutes of Health Osteoarthritis Biomarkers Consortium. Arthritis Rheumatol 2016, 68(10): 2422-2431. PMID: 27111771. https://doi.org/10.1002/art.39731*

The following table gives some demographic information about the participants with data currently released for the Project 22 data:

Project 22 sample: Distribution of Race by Sex

	White or Caucasian	Non-White	Total
Male	210	37	247
Female	265	88	353
Total	475	125	600

Project 22 sample: Distribution of Age by Sex

	Age (years)				
	45 to 49	50 to 59	60 to 69	70 to 79	Total
Male	25	92	66	64	247
Female	28	120	132	73	353
Total	53	212	198	137	600

3. Methods specific to Project 30

3.1 Image type

For this study, the sagittal and coronal IW TSE series, the sagittal 3D DESS WE, and the axial and coronal multiplanar reformats (MPRs) of the DESS series were used. For the MRI acquisition protocol, see the "MRI Manual" operation manual.

3.2 Time points

Baseline visit.

3.3 Measurement methods

Images were transferred to BICL for readings, which were performed blinded to the case/control status within the study. The images were assessed paired under the supervision of Dr. Ali Guermazi. Cartilage morphology, BMLs, osteophytes, meniscal damage, ACL/PCL tear, synovitis and effusion and extra articular features such as cysts and bursitis were scored. MOAKS scoring as described in sections 1.4 – 1.7 was performed.

3.4 Variables

See the dataset documentation file *kMRI_SQ_MOAKS_BICLxx_Contents.pdf* in the compressed documentation file for a complete list of all the variables in the dataset, their SAS variable names, descriptive variable labels and attributes.

3.5 Sample

Knees selected for reading in this study were part of a case/control study looking at patterns of OA related lesions in 200 knees with isolated lateral compartment radiographic knee osteoarthritis compared to 200 knees with isolated medial compartment radiographic osteoarthritis and to 200 knees with no radiographic osteoarthritis, matched on age and sex.

Further details can be found in this publication[3]:

- *Wise BL, et al. Magnetic resonance imaging lesions are more severe and cartilage T2 relaxation time measurements are higher in isolated lateral compartment radiographic knee osteoarthritis than in isolated medial compartment disease – data from the Osteoarthritis Initiative. Osteoarthritis & Cartilage 2017; 25(1) : 85-93.. PMID: 27539891 PMCID: PMC5182174* https://doi.org/10.1016/j.joca.2016.08.002

Project 30 sample: Distribution of Race by Sex

	White or Caucasian	Non-White	Total
Male	176	24	200
Female	284	89	373
Total	460	113	573*

*Race data missing for 1 participant

Project 30 sample: Distribution of Age by Sex

	Age (years)				
	45 to 49	50 to 59	60 to 69	70 to 79	Total
Male	21	71	60	49	201
Female	24	81	142	123	373
Total	45	155	202	172	574

4. Methods specific to Project 61

4.1 Image type
For this study, the sagittal and coronal IW TSE series, the sagittal 3D DESS WE, and the axial and coronal multiplanar reformats (MPRs) of the DESS series were used. For the MRI acquisition protocol, see the "MRI Manual" operation manual.

4.2 Time points
12-month, and 48-month visits.

4.3 Measurement methods
Images were transferred to BICL for readings, which were performed blinded to the clinical status of the participant. The images were assessed paired, with known chronological order, under the supervision of Dr. Ali Guermazi. MOAKS scoring as described in sections 1.4 – 1.7 was performed, with the following exceptions: (a) BML Size was scored on a 4 points scale 0: none, 1: < 25%, 2: 25-50%, 3: > 50% (b) # of BMLS and percent that was cystic were not assessed (c) osteophytes, effusion and inter-condylar/Hoffa's synovitis were not assessed, and (d) various extra-articular were not scored features (e.g.: bursitis, extra-articular cysts, etc)

4.4 Variables
See the dataset documentation file *kMRI_SQ_MOAKS_BICLxx_Contents.pdf* in the compressed documentation file for a complete list of all the variables in the dataset, their SAS variable names, descriptive variable labels and attributes.

4.5 Sample
Knees selected for this study were Kellgren and Lawrence Grade 0 in both knees, and their right knee MRIs were read (or left knee if right not available, or of poor quality). Further details are given in these publications[4,5]:

- *Sharma L et al. Knee tissue lesions and prediction of incident knee osteoarthritis over 7 years in a cohort of persons at higher risk. Osteoarthritis & Cartilage 2017, 25(7): 1068-1075. PMID: 28232012 PMCID: PMC5466844.* https://doi.org/10.1016/j.joca.2017.02.788
- *Sharma L et al. Clinical significance of worsening versus stable preradiographic MRI lesions in a cohort study of persons at higher risk for knee osteoarthritis. Ann Rheum Dis 2016, 75(9): 1630-1636. PMID: 26467570 PMCID: PMC4833701.* https://doi.org/10.1136/annrheumdis-2015-208129

Project 61 sample: Distribution of Race by Sex

	White or Caucasian	Non-White	Total
Male	377	38	375
Female	417	57	474
Total	754	95	849*

*Race data missing for 1 participant

Project 61 sample: Distribution of Age by Sex

	Age (years)				
	45 to 49	50 to 59	60 to 69	70 to 79	Total
Male	72	179	73	61	375
Female	67	175	148	85	475
Total	139	354	221	136	850

5. Methods specific to Project 63

5.1 Image type

For this study, the sagittal and coronal IW TSE series, the sagittal 3D DESS WE, and the axial and coronal multiplanar reformats (MPRs) of the DESS series were used. For the MRI acquisition protocol, see the “MRI Manual” operation manual.

5.2 Time points

Baseline, 12-month, 24-month, 36-month, and 48-month visits.

5.3 Measurement methods

Images were selected and prepared for reading by the Clinical Epidemiology Unit at Boston University under the supervision of Dr. David Felson. Images were transferred to BICL for readings, which were performed blinded to the clinical status of the participant. The images were assessed paired, with known chronological order, under the supervision of Dr. Ali Guermazi. MOAKS scoring as described in sections 1.4 – 1.7 was performed, with the following exceptions: (a) BML Size was scored on a 4 points scale 0: none, 1: < 25%, 2: 25-50%, 3: > 50% (b) # of BMLS and percent that was cystic were not assessed (c) osteophytes, effusion and inter-condylar/Hoffa’s synovitis were not assessed, and (d) various extra-articular were not scored features (e.g.: bursitis, extra-articular cysts, etc.)

5.4 Variables

See the dataset documentation file *kMRI_SQ_MOAKS_BICLxx_Contents.pdf* in the compressed documentation file for a complete list of all the variables in the dataset, their SAS variable names, descriptive variable labels and attributes.

5.5 Sample

This study was performed by the Clinical Epidemiology Unit at Boston University under the supervision of Dr. David Felson. Knees selected for this study were cases of incident radiographic OA, with controls with no radiographic OA and controls with radiographic OA (KLG=2) at baseline, matched to cases by age, sex and BMI. For this project, the variable READPRJ informs users about case/control status:

- 63A: case of incident radiographic OA by 12-month follow-up
- 63B: case of incident radiographic OA by 24-month follow-up
- 63C: case of incident radiographic OA by 36-month follow-up
- 63D: case of incident radiographic OA by 48-month follow-up
- 63E: control with no radiographic OA
- 63F: control with radiographic OA at baseline

Project 63 sample: Distribution of Race by Sex

	White or Caucasian	Non-White	Total
Male	209	23	232
Female	327	54	381
Total	536	77	613

Project 63 sample: Distribution of Age by Sex

	Age (years)				
	45 to 49	50 to 59	60 to 69	70 to 79	Total
Male	29	88	63	52	232
Female	41	122	137	81	381
Total	70	210	200	133	613

6. Methods specific to Project 65

6.1 Image type

For this study, the sagittal and coronal IW TSE series, the sagittal 3D DESS WE, and the axial and coronal multiplanar reformats (MPRs) of the DESS series were used. For the MRI acquisition protocol, see the “MRI Manual” operation manual.

6.2 Time points

Baseline, 12-month, 24-month, 36-month, and 48-month visits.

6.3 Measurement methods

This study was performed under the supervision of Dr. Kent Kwoh from the Arthritis Research Center at the University of Arizona (previously at the University of Pittsburgh Medical Center – one of the OAI Clinical Centers). Images were selected for reading by Dr. Kwoh and his team and were prepared and sent to BICL for readings which were done blinded to case/control status in the study. The images across all visits were assessed together, and with known chronological order, under the supervision of Dr. Ali Guermazi. MOAKS readings were performed as described in sections 1.4-1.7, with the exception that osteophytes weren’t scored.

6.4 Variables

See the dataset documentation file *kMRI_SQ_MOAKS_BICLxx_Contents.pdf* in the compressed documentation file for a complete list of all the variables in the dataset, their SAS variable names, descriptive variable labels and attributes.

6.5 Sample

Knees in this project are from the incident osteoarthritis and knee replacement (TKR) cohorts of the Pivotal OAI MR Imaging Analyses (POMA) study. The incident OA cohort consists of 710 knees selected for a nested, 1-to-1 matched case/control analysis. Cases were defined as knees that progressed to Kellgren-Lawrence (KL) grade ≥ 2 by the 48-month visit. Controls were defined as knees that did not have or progress to KL grade ≥ 2 by the 48-month visit. Matching was on participant age (within five years), sex, and KL grade in both knees at the baseline visit. Selections were performed using the OAI Outcomes99 version 6 dataset. The TKR cohort consists of 450 knees selected for a nested, 1-to-1 matched case/control analysis. Cases were defined as knees that underwent a primary total knee replacement after the baseline visit, but before the 60-month visit. Controls were defined as knees that did not undergo knee replacement prior to the 60-month visit. Matching was on participant age (within five years), sex, and KL grade in both knees at the baseline visit. Selections were performed using the OAI Outcomes99 version 6 dataset. Further details about this study are provided on the study webpage at https://www.niams.nih.gov/funding/Funded_Research/Osteoarthritis_Initiative/pivotal_mri.asp

The following are two publications[6,7] that describe the MOAKS readings from POMA:

- *Roemer F, et al. What comes first? Multitissue involvement leading to radiographic osteoarthritis: magnetic resonance imaging-based trajectory analysis over four years in the osteoarthritis initiative. Arthritis Rheumatol 2015, 67(8): 2085-2096. PMID: 25940308 PMCID: PMC4519416. https://doi.org/10.1002/art.39176*
- *Roemer F, et al. Can structural joint damage measured with MR imaging be used to predict knee replacement in the following year? Radiology 2015, 274(3): 810-820. PMID: 25279436 PMCID: PMC4455669. https://doi.org/10.1148/radiol.14140991*

Some knees were read as part of the incident OA sub-study and then later for the TKR sub-study of POMA. In that situation, the reading with the longer followup period was used in these datasets.

Project 65 sample: Distribution of Race by Sex

	White or Caucasian	Non-White	Total
Male	328	47	375
Female	517	140	657
Total	845	187	1032*

* Race data missing for 1 participant

Project 65 sample: Distribution of Age by Sex

	Age (years)				
	45 to 49	50 to 59	60 to 69	70 to 79	Total
Male	34	127	109	106	376
Female	54	234	234	135	657
Total	88	361	343	241	1033

7. References

1. *Hunter DJ et al. Evolution of semi-quantitative whole joint assessment of knee OA: MOAKS (MRI Osteoarthritis Knee Score). Osteoarthritis and Cartilage 2011; 19(8); 990-1002. PMID: 21645627 PMCID: PMC4058435* *http://dx.doi.org/10.1016/j.joca.2011.05.004*
2. *Collins JE et al. Semiquantitative Imaging Biomarkers of Knee Osteoarthritis Progression: Data From the Foundation for the National Institutes of Health Osteoarthritis Biomarkers Consortium. Arthritis Rheumatol 2016, 68(10): 2422-2431. PMID: 27111771.* *https://doi.org/10.1002/art.39731*
3. *Wise BL, et al. Magnetic resonance imaging lesions are more severe and cartilage T2 relaxation time measurements are higher in isolated lateral compartment radiographic knee osteoarthritis than in isolated medial compartment disease – data from the Osteoarthritis Initiative. Osteoarthritis & Cartilage 2017; 25(1) : 85-93.. PMID: 27539891 PMCID: PMC5182174* *https://doi.org/10.1016/j.joca.2016.08.002*
4. *Sharma L et al. Knee tissue lesions and prediction of incident knee osteoarthritis over 7 years in a cohort of persons at higher risk. Osteoarthritis & Cartilage 2017, 25(7): 1068-1075. PMID: 28232012 PMCID: PMC5466844.* *https://doi.org/10.1016/j.joca.2017.02.788*
5. *Sharma L et al. Clinical significance of worsening versus stable preradiographic MRI lesions in a cohort study of persons at higher risk for knee osteoarthritis. Ann Rheum Dis 2016, 75(9): 1630-1636. PMID: 26467570 PMCID: PMC4833701.* *https://doi.org/10.1136/annrheumdis-2015-208129*
6. *Roemer F, et al. What comes first? Multitissue involvement leading to radiographic osteoarthritis: magnetic resonance imaging-based trajectory analysis over four years in the osteoarthritis initiative. Arthritis Rheumatol 2015, 67(8): 2085-2096. PMID: 25940308 PMCID: PMC4519416.* *https://doi.org/10.1002/art.39176*
7. *Roemer F, et al. Can structural joint damage measured with MR imaging be used to predict knee replacement in the following year? Radiology 2015, 274(3): 810-820. PMID: 25279436 PMCID: PMC4455669.* *https://doi.org/10.1148/radiol.14140991*
